# FireLog: An open-source, low-cost system for temperature logging during wildland fires with high spatial and temporal resolution

**DOI:** 10.1016/j.ohx.2025.e00722

**Published:** 2025-11-13

**Authors:** Nipuna Chamara, Yufeng Ge, Sabrina Russo

**Affiliations:** a Department of Biological Systems Engineering, University of Nebraska-Lincoln, Lincoln, NE 68588, USA; b School of Biological Sciences, University of Nebraska-Lincoln, Lincoln, NE 68588, USA; c Center for Plant Science Innovation, University of Nebraska-Lincoln, Lincoln, NE 68588, USA

**Keywords:** Controlled burn, Wildland fire, Thermocouple, IoT Data Logger, Land management, Arduino

## Abstract

Measuring flame, air, and soil temperatures during wildland fires, including wildfires and controlled burns in land management contexts, is crucial for research and applications in fire ecology, safety, and management in a wide array of ecosystems, from grasslands to forests. However, open-source and commercial systems are needed for measuring and logging flame and air temperatures that are user-friendly, economical, modular, and customizable. This paper details the design, development, and validation of the FireLog system. Laboratory validation experiments demonstrated high measurement accuracy, with a minimum coefficient of determination (R2) of 0.98 and the highest observed root mean square error (RMSE) of 29.5 °C when compared with a Campbell Scientific data logger in furnace tests spanning 20–1000 °C. These results confirm the FireLog system’s precision, repeatability, and robustness under controlled conditions. Field deployment during prescribed burns further validated its operational performance, confirming its ability to record temperature dynamics reliably in active fire environments. FireLog represents a practical and scalable tool for researchers and practitioners in fire science and land management.

## Graphical abstract

**Figure:**
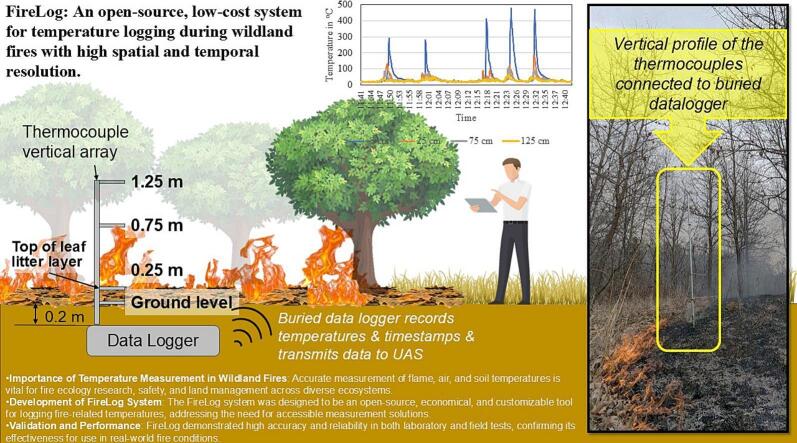

## 1 Hardware in context

### 1.1 Product requirement

The ability to measure and log temperatures during wildland fires is crucial for research, fire ecology, safety, and management in a wide array of ecosystems, ranging from grasslands to forests. Environmental studies in fire ecology focus on understanding the effects of fire on the ecosystem, including how flora, fauna, soils, and ecosystem services are affected by the intensity and behavior of wildland fires. For example, in fire-prone environments, serotinous plant species require specific temperature thresholds to be exceeded in order to trigger particular physiological processes in plants [1], such as seed release [2]. Additionally, fire properties also affect seed survival and germination [1]. Controlled (or prescribed) burns are used in many land management contexts, such as to reduce fuel loads to minimize the probability of wildfire and reduce potential wildfire damage, informing forest fire safety. Controlled burns are also used to control invasive species [2], reduce woody encroachment [3], and mimic natural fire regimes in fire-dependent ecosystems (e.g., oak savanna, long-leaf pine savanna in North America) [4,5,6]. They may also have effects on ecosystem properties, such as soil health and carbon storage [7]. Fire severity mapping, validating fire model behavior, and climate impact studies also rely on temperature measurements. Understanding, modeling, and predicting the responses of ecosystems to fire depend on thermal properties that are challenging to measure (Table 2). Therefore, to facilitate these activities, there is a strong demand for tools, technologies, and methods that can measure the absolute temperatures of flame, air, and soil during wildland fire with high spatial and temporal resolution and accuracy.

### 1.2 Related work

Satellite or airborne sensors, ground thermal cameras, infrared sensors, and thermocouples are used to measure fire temperature through thermal imagery [8]. While spectral imaging offers synoptic views, spectral temperature measurement methods have limitations because they measure relative, not absolute, temperatures and are influenced by atmospheric conditions such as smoke, soot, hot gases above the flame, and clouds. To obtain absolute temperatures from infrared sensors, it is essential to understand the emissivity of the material, which is particularly difficult for flames [9]. Furthermore, uncooled microbolometers exhibit higher error rates [10]. Although cooled microbolometers offer greater accuracy and sensitivity, they come at a significantly higher cost [11]. The iButton is a device that can measure temperatures below ground during fires, up to 140 °C [12]. However, they lack the necessary temperature range for above-ground fire temperatures. The thermocouple, particularly the type K thermocouple, has the advantage of measuring absolute temperature with high accuracy in ranges commonly observed during wildland fires (100–1000 °C [13,14]) without these limitations.

A substantial amount of literature is available regarding open hardware and proprietary equipment in fire temperature data logging systems. Commercial analog sensor data loggers include Campbell (CR1000x, Campbell Scientific, Inc., Logan, UT, USA), reported by [13,14,15,16,17], SPECTRUM data logger (VL1700, Veriteq Instruments, Inc., Richmond, British Columbia, Canada) reported by Mphale et al., [18], and HOBO data loggers (UX120-014 M & UX100-014 M, Onset Computer Corporation, Bourne, Massachusetts, USA) reported by [14,19,20,21,22] (Table 1). The common protocol for installing these data loggers to measure wildland fires is to enclose them in a waterproof bag or a commercial plastic enclosure and bury them under the soil or design a custom-made, heat-insulated box to protect them from high temperatures above ground. The most affordable commercial data logger on this list is the HOBO 4-Channel thermocouple logger, which costs approximately USD 360. Both the CR1000x and VL1700 are no longer available for purchase; however, the CR1000xe model has replaced the earlier CR1000 series data loggers. Another data logger from Campbell Scientific (Granite Temp 120, Campbell Scientific, Inc., Logan, UT, USA) is available and can support up to 20 thermocouples at a price of around USD 2,300.

**Table 1:** Specifications table.

Hardware name	FireLog
Subject area	• Engineering • Data storage hardware • Wildland fire ecology • Prescribed fire management • Land management • Plant ecology • Educational tools and open-source alternatives to existing infrastructure
Hardware type	• Field measurements and sensors
Closest commercial analog	• HOBO 4-Channel thermocouple data logger, HOBO • Granite Temp 120, Campbell Scientific • Campbell Scientific data loggers (e.g., CR1000x models)
Open-source license	• GNU General Public License version 3 (GPLv3) or later
Cost of hardware	• USD 208.18 (cost of data logger) • USD 438.13 (cost with five K-type thermocouples)
Source file repository	https://doi.org/10.17605/OSF.IO/YG2DU
OSHWA certification UID	US002738

**Table 2:** Thermal variables characterizing prescribed fire behavior and intensity to be quantified [10].

Thermal Variable	Description
Residence time	The duration a fire remains at a location.
Critical residence time	The duration a location exceeds the lethal temperature threshold for plant cells (60˚C)
Thermal AUC	Area under the temperature–time curve (AUC) at a location
Critical thermal	Thermal AUC for temperatures exceeding 60˚C
AUC Tmax	The highest temperature recorded at a location
Flame height	Maximum vertical extent of the flame above the ground

The advantages of these commercial products include longer battery life, high accuracy, compatibility with multiple types of thermocouples (B, E, J, K, N, R, S, and T thermocouple types), and reliability. However, their key disadvantage is the high cost of deploying them in replicated arrays with high spatial resolution [13], which is necessary for understanding the spatiotemporal dynamics of wildland fires, as well as the requirement for additional accessories such as batteries and enclosures. With real-time data monitoring available, the Axiom fire data logger (AEM, Germantown, Maryland, USA) provided a robust system for monitoring wildfire weather parameters using real-time satellite-based telemetry data reporting. Due to its large size, it is not feasible to use it in study contexts requiring portability. The price depends on the customization level defined by the user.

Development of an Arduino-based device for wildland fire detection and temperature data logging was reported by [13,23,24,25,26]. Integration of IoT technology was highlighted by [23,25,26,27]. These systems are more useful for fire monitoring and issuing fire alerts than for collecting scientific data. Moreover, only [13] reported that the Arduino-based system was capable of measuring temperatures beyond 200 °C, but no field evaluation was performed, which limits its application in wildland fires. Additionally, these systems can be further improved in terms of rapid field deployment, ergonomics (e.g., human–machine interfacing), and a modular design that supports all single-board computers in the Arduino MKR IoT family. Previous work designing Arduino-based IoT data loggers for crop and soil monitoring in agriculture informed the development of the FireLog system [31]. FireLog provides a more cost-effective and accessible solution, while enabling high-resolution spatial and temporal data logging for wildland fire applications.

The customized data logger (Table 3) presented in this paper was developed to monitor real controlled burns used in forest and grassland management. Still, it has many potential applications in other wildland fire contexts. It offers substantial improvements over existing systems by (1) reducing the cost of the data logger, enabling highly replicated study designs and larger scale data collection, (2) allowing eight thermocouples to be attached to a single node, enabling temperature data to be collected at fine spatial scales, (3) improving the ergonomics of quickly deploying the data loggers in the field, (4) having a modular design enabling customization to specific research and application needs, and (5) enabling future expansions in wireless data transmission.

**Table 3:** Core hardware specifications and operational characteristics of the FireLog temperature-logging system, including power supply, microcontroller, sensor interfaces, sampling capability, expected battery longevity, and known laboratory and field limitations, are included in Table 3.

Power Input	3.7 V Li-ion/LiPo battery (∼10,000 mAh) via JST; charging & config over waterproof USB 3.0 panel connector (5 V). Rocker on/off in series.
Microcontroller	Arduino MKR WiFi 1010 with ATSAMD21G18A (ARM Cortex-M0+) and u-blox NINA-W102 Wi-Fi/BLE module.
Supported Drivers/Buses	SPI (MAX31855/31856 thermocouple amp; microSD; SPI Flash), I^2^C/Qwiic (DS3231 RTC), GPIO (status LEDs), USB CDC serial, Wi-Fi (2.4 GHz) for NTP time sync, BLE for pre-deployment health check. (Future-ready swaps: MKR1300/1310 for LoRa, MKR1500 for cellular/NB-IoT, satellite modem via UART/SPI.)
Board Communication Method	Sensors: SPI (thermocouples), I^2^C (RTC). User I/O: USB serial, short-window Wi-Fi & BLE (setup/health). Data offload: microSD. No real-time telemetry in this study.
Hardware/PCB Dimensions	29 × 19 × 14 cm
Features	0–1000 °C range; stated precision ± 0.5 °C; 0.25 °C resolution; up to 8 K-type thermocouples; max sampling 0.5 Hz (1 sample/2 s with all 8 sensors); battery life up to ∼5.3 days at 2-s logging (and up to ∼7 days at 100-s); USB charging; external status LEDs; modular, IoT-ready design; buried deployment for prescribed burns.s
Hardware limitations	Max measurable temperature − 1000 °C Minimum resolution − 0.25 °C Sensor channels − Up to 8 K-type thermocouples Sampling frequency − Data collection frequency for one sensor is 4 Hz Deployment − Enclosure must be buried for prescribed-burn measurementsBattery life (max load) − Battery longevity will be around 5 days for the highest frequency
Field test limitations	Replication − Single prototype / limited replication Site/conditions − Single site & burn type Telemetry − Post-hoc data retrieval only Installation − Installation constraints: Logger must be buried (≥10–20 cm, deeper for hotter fires) and the pole sunk ≥ 30 cm
Laboratory test limitations	Calibration − No formal dry-block calibration performed Method − Furnace-based comparison only
Supported Peripherals	Expansion-ready peripherals (drop-in with minimal changes) LoRa: Swap MKR 1010 → MKR 1300/1310 for long-range links.Cellular / NB-IoT: Swap to MKR 1500 (3G/4G/LTE/NB-IoT) .Satellite: External modem (e.g., Iridium Certus dev kit) via UART/SPI. Additional sensors: Any I^2^C/SPI/UART/analog peripheral (e.g., soil-moisture, additional thermocouple types) supported by MKR I/O may require firmware additions and power budgeting.

### 1.3 User requirements

Flame heights, temperatures, and rate of movement are highly variable in wildland fire, and fire intensity and behavior can vary in complex ways related to fuel load, topography, and weather conditions [10]. Fire research and applications require high-accuracy and high-frequency measurements of flame, air, soil, and fuel (e.g., woody debris) temperatures at various heights above and below ground at a location, potentially over a few days, to capture this variation. Our sensor is optimized to collect data in such applications. The particular controlled burn application for which this sensor system was developed required measurements of temperature at heights of ∼3 cm (ground level in the leaf litter), 25 cm, 75 cm, and 125 cm above ground, with an accuracy of ±2 °C. However, any user can use the FireLog system to measure temperature at any desired height above or below ground, limited only by wire length and the support structure for the thermocouple. The response time of the type-K thermocouple in FireLog refers to how quickly the sensor detects changes in temperature. In field testing, data were collected at 5-second intervals, although the system supports a maximum sampling frequency of 0.5 Hz, which is appropriate for capturing faster fire dynamics.

FireLog was deployed to investigate how fire intensity and behavior influence juvenile tree survival during prescribed burns, requiring high levels of spatial replication. Therefore, the system needed to be cost-effective to deploy in large quantities at the desired spatial resolution, depending on the landscape context. The temperature values needed to be securely stored with timestamps for later data visualization, analysis, and modeling. The device should be user-friendly, indicate errors, operate for at least two days on a single battery charge, withstand waterlogging and high-heat conditions, and be securely positioned during the data collection process. The user must have the option to verify the device's data collection conditions after installation, as prescribed fires are logistically difficult and costly to implement.

## 2 Hardware description

Building on previous open-source developments [13,23], this study employs Arduino MKR boards, which are specifically designed for low-power IoT applications, replacing the earlier Arduino UNO and MEGA development boards. This new hardware specified in the Table 3 is compact, requires significantly less space, and is power-efficient, allowing the system to run for longer periods. It features extensive I/O connectivity, including digital, analog, pulse width modulation (PWM), I2C, SPI, and UART options, along with built-in support for Wi-Fi, Bluetooth, LoRa, and NB-IoT. Additionally, we have introduced an off-the-shelf thermocouple shield manufactured by Neem Tech LLC, which can read up to eight thermocouples. This integration enables us to utilize an enclosure that is easily buried shallowly in the soil, thereby protecting the unit from heat damage.

On the outside of the enclosure, a waterproof on/off switch is located, allowing the user to turn off the power supply when it is not in use. Two waterproof LED indicators display the system's operational status to the user without requiring the enclosure to be opened. A red LED indicates any issues in the system, while a green LED signals successful data recording. A waterproof USB Type A female USB 3.0 connector enables the user to interact with the serial data output from the data logger, allowing for troubleshooting of errors and data downloading. The lowest IP protection rating is for the LED lights (IP65); hence, we rate the overall device's IP rating as IP65. FireLog can be recharged through the same USB connector, a significant improvement over battery swapping [21] and additional power converter circuits [24], thereby enhancing FireLog's user-friendliness. The fully charged battery can last up to 7 days with data collection at every 100 s, and can last up to 5.3 days when data collection is at every 2 s.

Additionally, users can configure the Wi-Fi hotspot name and password through the USB connection. Wi-Fi connectivity enables the system to synchronize the data logger's time with network time, thereby enhancing data accuracy and ensuring consistency in timestamps across multiple units. In the field, users can connect to the FireLog via Bluetooth to check the timestamp accuracy and functionality before burying it. All Wi-Fi connectivity and Bluetooth activities shut down after 2 min to conserve battery life. Still, data collection starts automatically when the unit is turned on. Users can restart the FireLog to regain access to settings through serial or Bluetooth communication. There is no need for real-time data transmission in controlled burn fire monitoring, as the users are aware of the burn locations and start timing. Additionally, the safe distance required during prescribed fires exceeds the range of Wi-Fi and Bluetooth data transmission. Therefore, no effort was made in this study to facilitate real-time data transmission.

FireLog’s current design enables users to upgrade the system for LoRa or mobile connectivity without major modifications, as the Arduino MKR family maintains the same form factor.

The K-type thermocouple used in FireLog features high-fiber insulation. It is 2.5 m long, allowing the user to measure temperatures at heights of approximately 2 m above or below the ground. K-type thermocouples are available in lengths up to 5 m. The thermocouples are supported by a 19-mm-diameter galvanized steel pipe using a cable clamp; however, any fireproof support structure could be employed. The thick wire of the thermocouple allows it to extend at least 25 cm from the support. Eight thermocouples can connect to the data logger; in our application, we utilized two sets of four thermocouples at 0 cm, 25 cm, 75 cm, 100 cm, and 125 cm heights. Although the thermocouple can measure temperatures ranging from −50 °C to 1000 °C, the thermocouple breakout board we used supports readings from −200 °C to 1350 °C with a 0.25 °C resolution.

The total cost for custom hardware, excluding the thermocouple, is USD 208.18. This option is significantly lower in cost, supporting four additional thermocouples and offering more customizable features compared to the proprietary data logger with the lowest cost (USD 360, HOBO 4-channel thermocouple data logger). The cost-effectiveness of this unit enables users to deploy data loggers to more locations at their study site, recording vertical temperature profiles and thereby enhancing the spatial resolution of temperature measurements during wildland fires. The total cost for the data logger, housing, and four thermocouples, which comprise a single FireLog unit, is USD 438.13.

**The key features of the FireLog system are:**

• Measures temperatures from 20 °C to 1000 °C with high precision (±0.5 °C) and a maximum sampling rate of 0.5 Hz, enabling replication suitable for wildland fire applications.
• IP65-rated waterproof housing with external status indicators.
• Customizable data collection through programmable software.
• A simple, reliable, and portable design that leverages cost-effective, off-the-shelf open-source hardware and software.
• The modular design with IoT connectivity supports various communication technologies, including LoRa (e.g.: Replace MKR 1010 with Arduino MKR 1300/1310), mobile communication (3G, 4G, LTE, narrow band IoT) (e.g.: Replace MKR 1010 with Arduino MKR 1500), and satellite (e.g.: Iridium Certus 9704 Satellite IoT Developer Kit), facilitating further enhancements.
• Improved user-friendliness in operating FireLog with both serial connectivity and mobile connectivity via Bluetooth.
• Compatibility with other sensors without significantly altering field data collection (e.g., leaf temperature using a type T thermocouple, soil moisture).
• Battery life of up to 5.3 days at a 0.5 Hz sampling rate.

## 3 Design files

### 3.1 Design files summary

The **Hole cut enclosure DL001.pdf** contains the recommended hole sizes and the reference X and Y distances for each hole (Table 4).

**Table 4:** Design files summary.

Design file name	File type	Open source license	Location of the file
Hole cut enclosure DL001.pdf	2D CAD	GPLv3	https://doi.org/10.17605/OSF.IO/YG2DU
DIN rail mounted enclosure DL002.pdf	2D CAD	GPLv3	https://doi.org/10.17605/OSF.IO/YG2DU
PCB mounted enclosure DL003.pdf	2D CAD	GPLv3	https://doi.org/10.17605/OSF.IO/YG2DU
PCB mounted enclosure DL003.SLDASM	3D CAD	GPLv3	https://doi.org/10.17605/OSF.IO/YG2DU
Final assembly and parts file.zip	3D CAD	GPLv3	https://doi.org/10.17605/OSF.IO/YG2DU
Power supply management circuit files.zip	PCB Layout	GPLv3	https://doi.org/10.17605/OSF.IO/YG2DU
Thermocouple data logger program V10.2.c	.ino file (Arduino sketch file)	GPLv3	https://doi.org/10.17605/OSF.IO/YG2DU

**DIN rail mounted enclosure DL002.pdf** provides the location of the DIN rail mounting within the enclosure (Table 4).

The file PCB mounted enclosure DL003.pdf provides placement details for the components secured within the enclosure (Table 4).

The file PCB mounted enclosure DL003.SLDASM is the SolidWorks assembly used to generate all design drawings. The associated 3D part files were obtained from manufacturers’ websites (Table 4).

The **Final assembly and parts file.zip** contains all the assembly files and part files used in the SolidWorks design, organized in a zipped folder (Table 4).

The **Power supply management circuit files.zip** was designed using the open-source KICAD software. It includes the circuit schematic diagram along with the printable PCB file. Future iterations of this design can be executed without printing the specific PCB, but by using a PCB dot breadboard (Table 4).

The file Thermocouple data logger program V10.2.c is the latest version of the custom software developed by the authors and includes all features described in this manuscript (Table 4).

## 4 Bill of materials

In this section, we have listed the components needed to reproduce the data logger. All items were sourced from U.S.-based suppliers, and costs are reported in USD. Only the homepage of each vendor is provided; specific components can be located using the names given in Table 5. Items made from multiple materials are categorized as composite in the Table 5. Some parts were received at no cost, along with the main items (e.g., DIN rail with the enclosure); therefore, prices are not mentioned for these additional components (e.g., DIN rail, enclosure screws, grid plate).

**Table 5:** Bill of materials summary.

Part ID	Component	Quantity	Unit cost (US$)	Total cost (US$)	Supplier link	Material type
Part #1	Electronics enclosure box	1	31.00	31.00	https://shorturl.at/QslJP	ABS plastic
Part #2	Arduino MKR 1010	1	38.00	38.00	https://shorturl.at/nnsyx	Semiconductor, metal, and composite
Part #3	Breakout for Arduino MKR	1	20.00	20.00	https://shorturl.at/FUiDt	Composite
Part #4	Thermocouple breakout board	1	43.00	43.00	https://shorturl.at/Pk73x	Composite
Part #5	Lithium polymer ion battery	1	15.00	15.00	https://shorturl.at/hqeE7	Composite
Part #6	Arduino MKR MEM Shield	1	23.00	23.00	https://shorturl.at/WE8Hn	Composite
Part #7	Round rocker switch	1	1.50	1.50	https://shorturl.at/cJmNf	Composite
Part #8	LED	2	2.25	4.50	https://n9.cl/8uj0b	Polycarbonate/semiconductor
Part #9	Precision (real-time clock) RTC	1	14	14.00	https://www.adafruit.com/product/5188	Composite
Part #10	Qwiic Cable	1	1.50	1.50	https://www.adafruit.com/product/4210	Composite
Part #11	Coin cell battery	1	0.95	0.95	https://www.adafruit.com/product/380	Composite
Part #12	Micro USB Cable	1	1.29	1.29	https://n9.cl/9rruz	Composite
Part #13	USB 3.0 waterproof connector	1	6.00	6.00	https://n9.cl/8n6rf	Polymer
Part #14	Cable gland	1	0	0	Associated with Part #1	Polymer
Part #15	DIN rail	1	0	0	Associated with Part #1	Metal
Part #16	DIN rail mounting adapter	4	0.75	3.00	Associated with Part #1	Polymer
Part #17	DIN rail mount self-tapping screws	4	0	0	https://n9.cl/opvub	Metal
Part #18	JST-XH connector	1	0.34	0.34	https://n9.cl/9ik3po	Metal/polymer
Part #19	Resistors	2	0.06	0.12	https://n9.cl/kbivju	Semiconductor
Part #20	PCB prototype board	1	0.22	0.22	https://n9.cl/kwsj79	Composite
Part #21	K-Type Thermocouple	5	39.99	199.95	https://n9.cl/816ss0	Composite
Part #22	PCB standoff (A 600-unit standoff kit costs)	1	15.00	∼0	https://n9.cl/ppyz15	Polymer
Part #23	SD Card	1	2.5	2.50	https://n9.cl/a9xppm	Metal/polymer
Part #24	Male to female jumper cables	20	0.05	1.00	https://n9.cl/q96ury	Metal/polymer
Part #25	GI pipe	1	25.96	30.00	https://n9.cl/e78osb	Metal

## 5 Build instructions

We have sourced all components from online stores. If the engineering specifications are met during production, these parts can be replaced with compatible items. The first step is to drill six holes on one side of the electronic enclosure box (Part #1) to install the round rocker switch (Part #7), USB 3.0 connector (Part #13), LED lights (Part #8), and cable glands (Part #14). The hole specifications table (Table 6) outlines the hole locations and diameters. After drilling the holes, remove any remaining plastic fragments from the edges and smooth them with sandpaper before installing parts #7, #8, #13, and #14. This cleaning process ensures that the parts are securely mounted and properly aligned with the enclosure body. The 3D CAD file and 2D drawing provide the dimensions for further reference. Depending on the size of the enclosure used, the hole diameters and positions can be altered.

**Table 6:** Hole specifications table (reference is the bottom left corner of the data logger).

Tag	X position (mm)	Y position (mm)	Diameter (mm)
Hole1	60	80	12
Hole2	100	80	12
Hole3	140	80	13
Hole4	30	25	29
Hole5	80	25	22
Hole6	140	25	13

In step two, attach the DIN rail (Part #15) to the grid mount using the included DIN rail mount screws (Part #17). The mounting dimensions are specified in the design file drawing #DL002. These dimensions can be adjusted depending on the size of the enclosure being used.

Next, 5 DIN rail mounting adapters can be affixed onto the DIN rail, enabling the user to adjust the spacing to match the Arduino MKR breakout board (Part #3), the thermocouple shield (Part #4), and the precision RTC (Part #9) on a PCB standoff (Part #22). The arrangements for Parts #3, #9, and #22 can be found in DL003. The LiPo battery (Part #5) can be secured to the dot plate using double-sided tape.

Once the Arduino MKR breakout board is mounted on the DIN rail mounting adapters, the Arduino MKR1010 and MKR MEM shield can be stacked by aligning the pins on each board. The MEM shield should be positioned on top for easy access to the SD card when data needs to be downloaded.

**Fig. 1:**
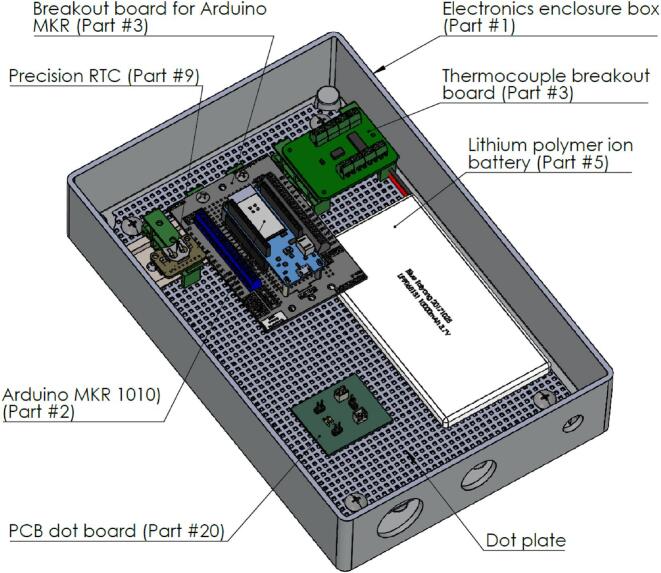
Isometric view of the data logger design after mounting all the PCBs.

**Fig. 2:**
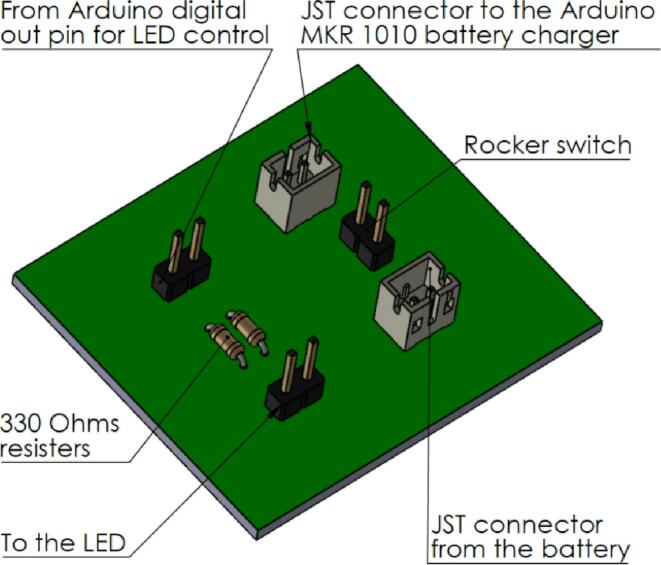
Custom-built power supply control circuit.

We created a separate PCB (Fig. 2) using the PCB dot boards to add an on/off switch to the system. The rocker switch (Part #7) is in series with the battery (Part #5). Additionally, 330 Ω resistors are connected in series with the LED bulbs to reduce the current flow through the LEDs. The isometric view in Fig. 1 illustrates the assembly described here.

The schematic diagram titled “Power supply management circuit files.zip” in the design file provides the circuit diagram drawn using the KiCAD open-source software. The main function of this circuit is to cut off the power to the Arduino MKR1010 SBC. Since the Arduino MKR1010 does not have an on/off switch, it is essential to keep the FireLog turned off when not in use. In future reproductions, we recommend adding a rocker switch or push button to trigger the start and stop of data collection as needed. This will allow the user to control the data collection while the FireLog is on, saving battery energy.

**Fig. 3:**
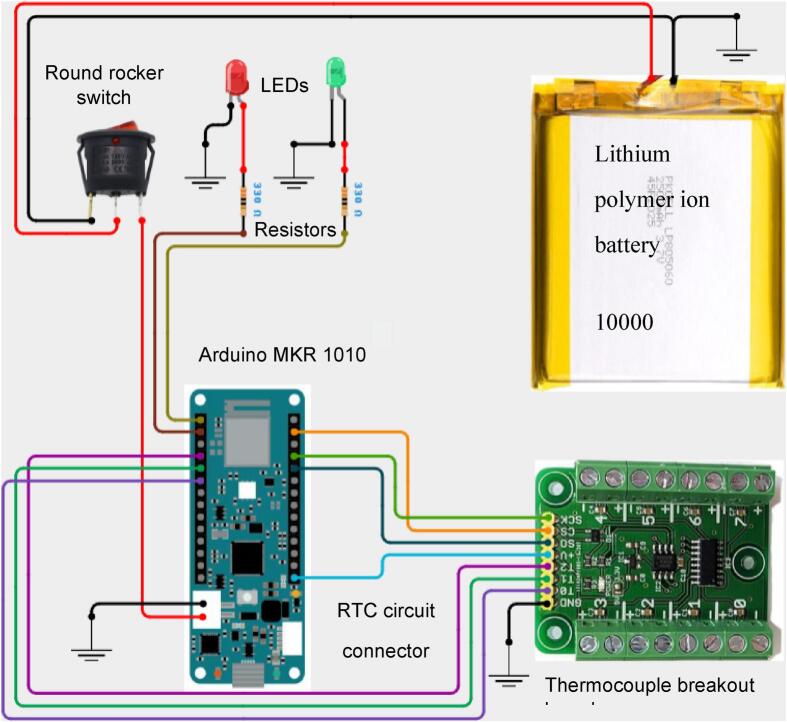
Wiring diagram of the data logger (red wire − +V, green wire − ground). (For interpretation of the references to colour in this figure legend, the reader is referred to the web version of this article.)

Fig. 3 was designed using the https://app.cirkitdesigner.com online tool illustrates the wiring schematic of the custom thermocouple data logger system. Table 7 provides a detailed description of the wiring diagram. Fig. 2 was created using the KiCAD web app. The thermocouple interface utilizes SPI communication, with MISO (Master In/Slave Out), SCK, and CS lines connected to MISO, SCK, and D7 on the MKR board, respectively. Channel selection is achieved through digital pins D0 to D2 on the MKR 1010 board (T0 to T2 in the thermocouple shield). LED indicators are connected through current-limiting resistors and controlled via D5 (red) and D6 (green). A rocker switch manages power delivery from the 3.7 V, 10,000 mAh LiPo battery via a JST connector. All components share a common ground through the thermocouple breakout board GND rail. This configuration provides a portable and efficient solution for high-resolution temperature monitoring in field applications. The USB cable connects from the MKR 1010 to the USB connector on the enclosure. The thermocouples can be passed through the cable gland, and by using reusable and non-drying modeling clay, the gland hole can be further sealed to prevent water leakage.

**Table 7:** Wiring connections for the FireLog data logger.

Signal/Component	Connected To	Notes
**Ground Connections**		
LED (red) cathode	MAX31855 GND	Shared ground reference
LED (green) cathode	MAX31855 GND	Shared ground reference
Arduino MKR Wi-Fi 1010 GND	MAX31855 GND	Common GND

**Thermocouple breakout board**	**Breakout board for Arduino MKR 1010**	**Thermocouple SPI communication**
T0	D0	Thermocouple channel select/control
T1	D1	Thermocouple channel select/control
T2	D2	Thermocouple channel select/control
MISO (Master In/Slave Out)	MISO	SPI data out from MAX31855
SCK (Serial clock)	SCK	SPI clock
CS (Chip select)	D7	SPI chip select
Power	5 V	Power supply to MAX31855 breakout

**LEDs and Resistors**		**Current-limiting and control**
Resistor (1) pin1	LED (green) anode	Series resistor for green LED
LED (red) anode	Resistor (2) pin1	A series resistor for the red LED
Resistor (1) pin2	Arduino D6	Digital control for green LED
Resistor (2) pin2	Arduino D5	Digital control for the red LED

**Power connections (Battery + Switch)**		LIPO & switch wiring
LIPO Battery +	Rocker Switch Input +	Rocker switch input side
Rocker Switch Output +	JST 2-pin connector pin 2	Power supply to the FireLog
LIPO Battery –	Rocker Switch Ground	Common negative
Rocker Switch Ground	JST 2-pin connector pin 1	Negative to the board

The precision RTC board (Part #9) connects to the 5-pin I2C port on the Arduino MKR 1010 via a 5-pin to 4-pin Qwiic Cable (Part #10). A coin battery (Part #10) must be installed in the RTC to ensure it keeps time when the Arduino MKR 1010 is not operating.

## 6 Operation instructions

**Fig. 4:**
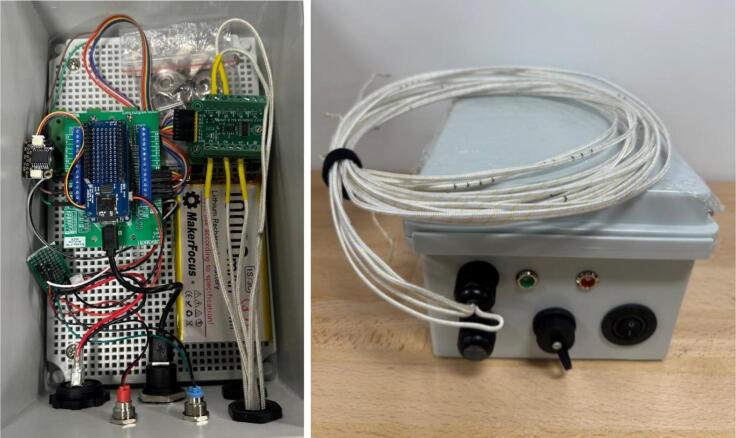
Assembled data logger internal view (left) and the HMI (Human Machine Interface) panel view (right).

After assembling the system (Fig. 1, Fig. 4), the data logger is ready for data acquisition and processing. It can be charged and programmed through the USB 3.0 connector when the rocker switch is in the “ON” position. The Thermocouple data logger program, version 10.2, can be uploaded using the Arduino IDE software, which allows FireLog to be set for charging. Like a typical Arduino program, we first defined all the required libraries and instances and declared the variables initially. Next, the setup loop runs once and then moves to the infinite loop, which handles data reading and storage (6). Once charged, if FireLog is being used for the first time, the Wi-Fi SSID, password, and data collection frequency must be configured via the USB connection. Any serial data communication software on a computer can be used for this purpose. This option will be available for up to 20 s after restarting FireLog; however, we have only used the Arduino IDE serial data terminal.

**Fig. 5:**
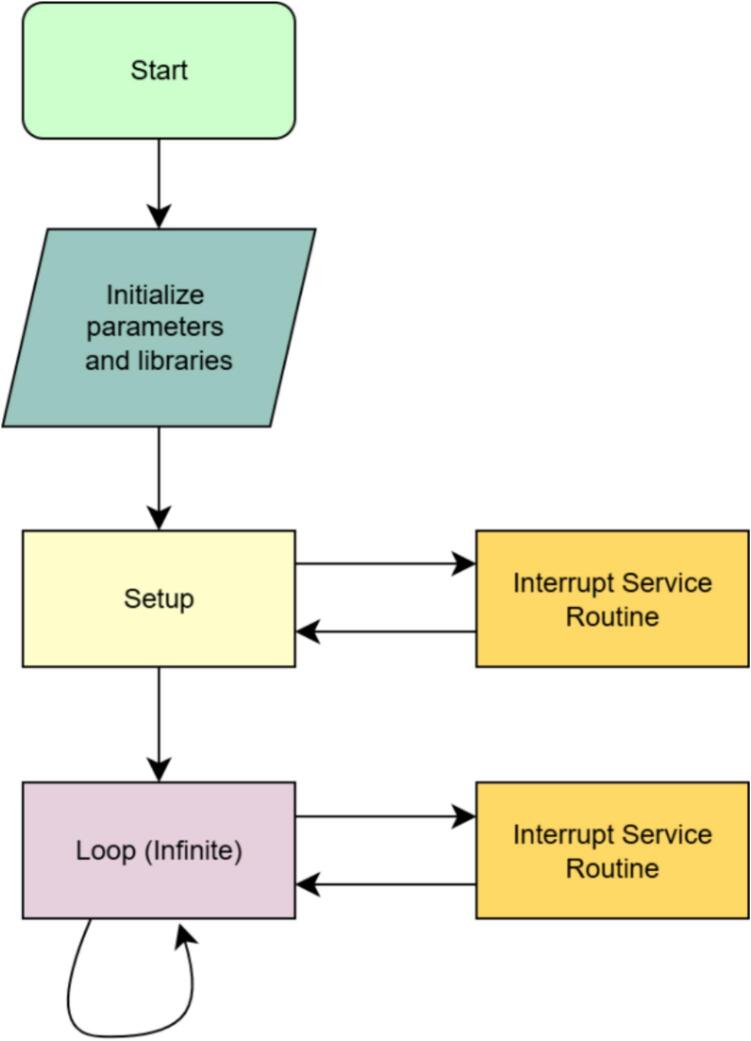
Flowchart of the FireLog data logger program (version 10.0).

**Fig. 6:**
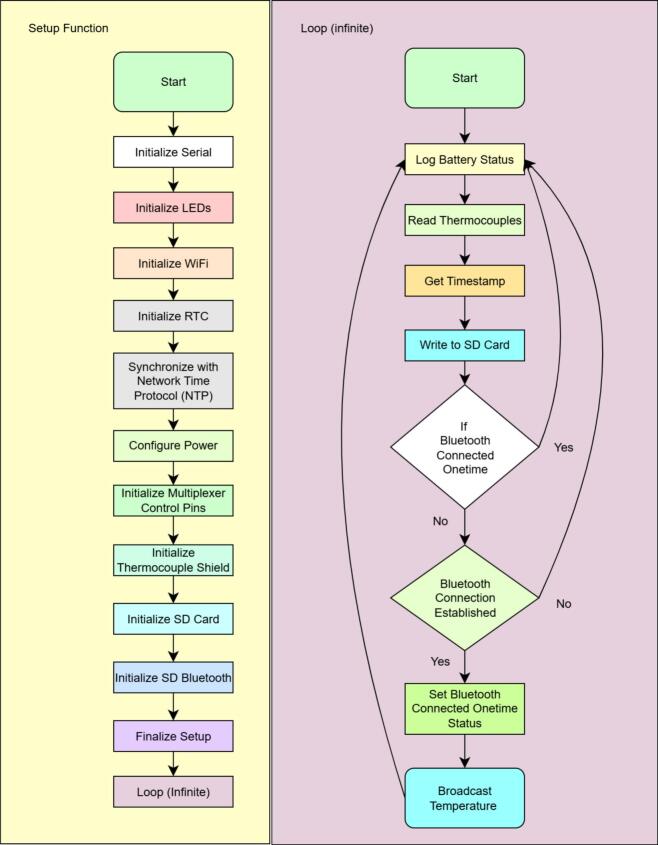
Detailed flow chart of the setup function and the infinite loop function in the arduino program (version 10.0).

Once the SSID and password are set, FireLog will connect to the appropriate Wi-Fi network and update the date and time on the RTC (Part #9). This step is essential for maintaining the accuracy of the RTC time. After updating the date and time, the Arduino MKR 1010 will turn off Wi-Fi to conserve battery energy by eliminating the power demand required to keep Wi-Fi active. Likewise, the Bluetooth connection is activated only once to allow the user to check.

the status of the thermocouple readings and the timestamp accuracy. Fig. 5 and Fig. 6 outline the data logger program version 10.0.

This process is generally helpful for identifying any abnormalities in the FireLog system just before deployment in the field. The Bluetooth connection will be active once FireLog powers on (Fig. 6), helping to conserve battery energy. The LightBlue® iOS app, developed by Punch Through Design LLC, is the app we have tested. However, there are generally free apps available for both iOS and Android mobile operating systems that support Bluetooth FireLog scanning and connection (e.g., Bluetooth for Arduino app, Dabble app).

**Fig. 7:**
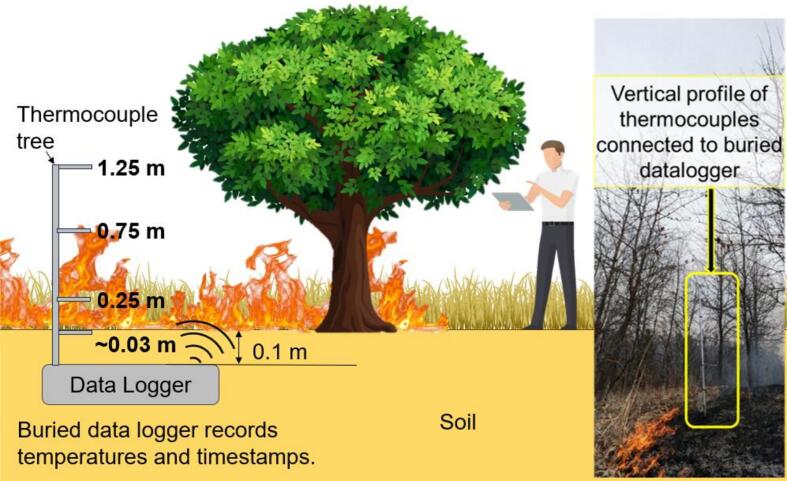
Schematic of FireLog field installation, illustrating buried data logger placement and vertical profile of thermocouples attached to a support pole.

Once these steps are complete, the FireLog is buried before controlled burning begins. The depth should be at least 10 cm for light-controlled burns and should increase depending on the fuel load and anticipated fire intensity. In our data collection (Fig. 7), the thermocouples were attached to a galvanized iron pole at heights of ∼3 cm (ground layer in the leaf litter), 25 cm, 75 cm, and 125 cm. The pole will usually have adequate stability if sunk to a depth of at least 30 cm in the soil, depending on the soil type. Fig. 7 illustrates the field installation. After the fire front passes the data logger and the thermocouple tree, it can be accessed safely. FireLog can be retrieved from the field, and the data can be copied from the SD card using an SD card reader. The data is saved on the SD card with a timestamp, and the file name will be the date of data collection. One safety tip is to avoid touching the thermocouple tree once the firefront has passed, as it will still be hot and therefore, unsafe to touch.

## 7 Validation and characterization

Laboratory validation of the FireLog system was conducted to assess its accuracy and consistency relative to a standard reference device. A sensor calibration was not performed due to the unavailability of the standard temperature sensor calibration instruments. The validation utilized a Campbell Scientific data logger equipped with two K-type thermocouples as the reference system. The FireLog system was set with eight K-type thermocouples. Both systems were placed near the Thermo Scientific Thermolyne Benchtop Industrial Muffle Furnace (Hogentogler & Co. Inc., Columbia, MD, US) to create a 1000 °C environment to test the FireLog along with the Campbell data logger. All the thermocouples, including 8 thermocouples from FireLog and 2 thermocouples attached to the Campbell Scientific data logger, were bundled using steel wires 2 in. away from the thermocouple hot junction. The thermocouples were placed in a non-contact arrangement, but in proximity to each other. Next, the bundle of thermocouples was inserted into the furnace and positioned above the furnace bed, ensuring that the hot junctions of any thermocouples did not come into contact with the furnace bed or its inner walls.

**Fig. 8:**
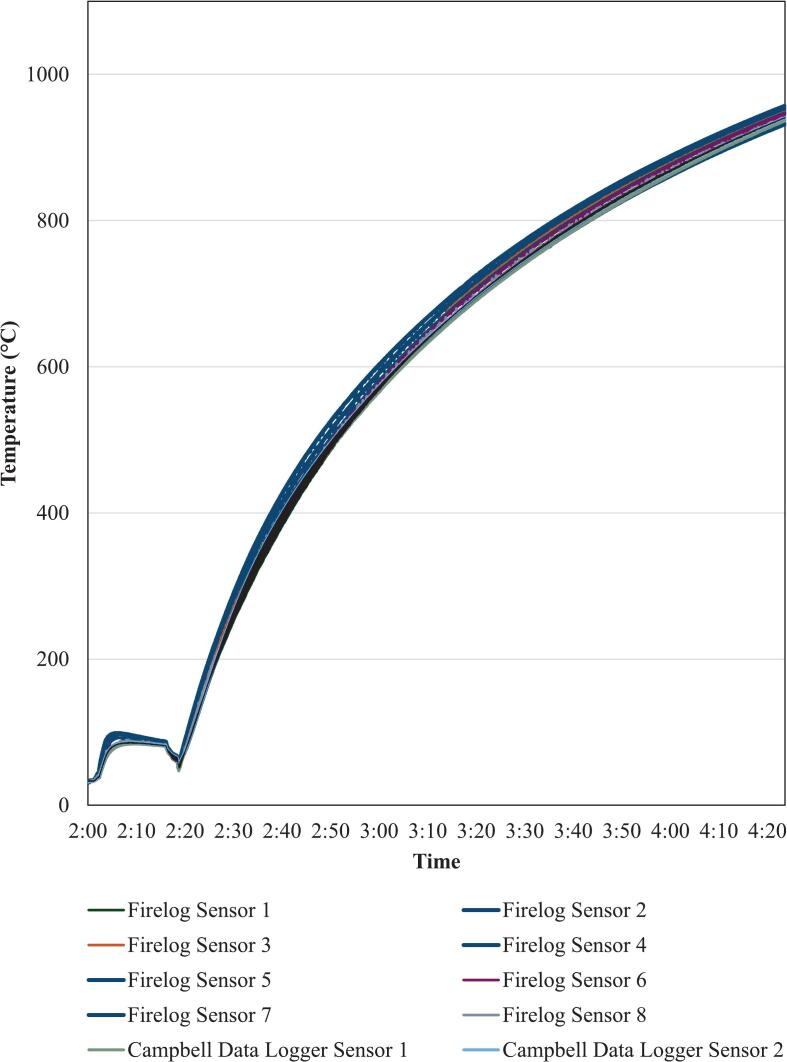
Temperature profiles recorded by type K thermocouples using the FireLog system (Sensors 1–8) and the Campbell Scientific CR300 data logger (Sensors 1–2).

**Fig. 9:**
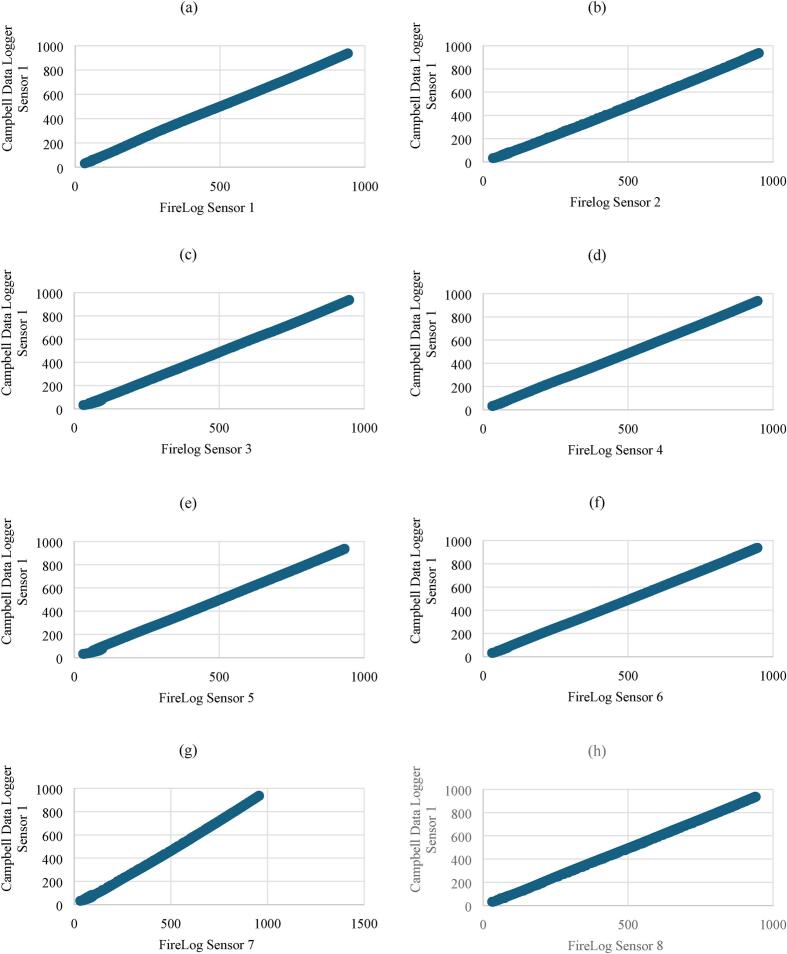
Pairwise linear regression analysis of temperature data recorded by Type K thermocouples using the FireLog data logger (Sensors T1–T8) compared with the Campbell Scientific CR300 data logger (Sensor 1-CD T1). Panels show: (a) CD T1 vs FL T1, (b) CD T1 vs FL T2, (c) CD T1 vs FL T3, (d) CD T1 vs FL T4, (e) CD T1 vs FL T5, (f) CD T1 vs FL T6, (g) CD T1 vs FL T7, (h) CD T1 vs FL T8.

The furnace was operated at maximum heating. After 15 min, heating was paused to confirm that the thermocouples remained securely positioned. Once verified, heating was resumed until the furnace reached 1010 °C, at which point it was shut down. Fig. 8 shows the temperature records of all 10 thermocouples. The Campbell data logger was only able to measure temperatures up to 949 °C, while FireLog recorded up to 1016 °C, exceeding the Campbell data logger's maximum logging temperature. All regression models exhibited coefficients of determination (R2) exceeding 0.98 (Fig. 9), indicating a strong linear relationship and suggesting high consistency in temperature trends between sensors even at this high temperature range. Maximum RMSE reported by the sensor at the 1st port was 29.5 °C, while the relative error was less than 3.7 %.

These findings support the reliability of the FireLog system in reproducing temperature profiles consistent with those recorded by the Campbell Scientific data logger, reinforcing its potential suitability for high-temperature monitoring applications.

The standard temperature sensor comparison calibration required a dry block calibrator. [28]. As a calibrator was unavailable, validation was conducted using a furnace, which is subject to fluctuations from convection currents, radiation imbalance, and larger chamber volume. In contrast, dry block calibrators employ high-conductivity metals that maintain stable, uniform temperatures. On the other hand, the dry block calibrator consists of high-conductivity metals that ensure stable and uniform temperatures across the dry block well. Therefore, we assume the limitations of the furnace-based temperature comparison caused the small fluctuations reported during the validation experiment.

To assess the operational longevity of the FireLog datalogger under different sampling regimes, a controlled battery life experiment was conducted. A total of nine FireLog units, each equipped with a 10,000 mAh lithium-ion battery, were fully charged prior to deployment. The devices were programmed to record data at three distinct logging intervals: every 2 s, every 10 s, and every 100 s. According to Table 8, the 2-second interval units averaged 5.3 days of runtime, while the 10-second interval units lasted 6.9 days. The 100-second interval units exhibited an average runtime of 7.0 days. These results suggest that reducing the logging frequency provided only modest improvements in battery longevity, with diminishing returns at lower sampling rates.

**Table 8:** Summary of FireLog battery longevity results under varying sampling intervals.

Logger ID (Sampling Interval)	Start Time	End Time	Battery Life (Days)
FireLog 1 (2 s)	08/27/2025 20:57	09/01/2025 08:30	4.5
FireLog2 (2 s)	08/27/2025 20:57	08/31/2025 16:00	3.8
FireLog4 (2 s)	08/27/2025 20:32	09/02/2025 14:30	5.7
FireLog8 (2 s)	08/27/2025 20:37	09/03/2025 7:37	6.5
FireLog6 (10 s)	08/27/2025 20:17	09/03/2025 15:56	6.8
FireLog7 (10 s)	08/27/2025 20:55	09/03/2025 20:53	7.0
FireLog9 (10 s)	08/27/2025 20:14	09/02/2025 16:35	5.8
FireLog1 (100 s)	08/27/2025 21:13	09/03/2025 14:11	6.7
FireLog3 (100 s)	08/28/2025 12:21	09/06/2025 00:09	8.5

The maximum voltage of the batteries when fully charged ranged from 3.6 V to 3.9 V, and the significantly different charge retention times highlight the lack of standardization among the batteries. Further studies are required to determine whether this result is a limitation of the rechargeable battery used in this development.

Additionally, there is a need to optimize the ideal current-consuming circuits, such as the LED driver circuit. We assume the reason for the shorter battery life in the 100-second interval device compared to the 2-second interval device is that the LED driver circuit's power consumption is high.

The intended application of this data logger is to measure the temperatures of controlled burn fires using thermocouples. On March 14, 2024, a field deployment was conducted to demonstrate the use of the developed data logger for controlled burning on private property near the township of Rulo in southeastern Nebraska, USA. Only one prototype was built at this time. A controlled burn was implemented in an oak woodland with dry leaf litter and fine woody debris on the ground layer, using a backing fire (i.e., fire movement is against the wind). We buried the data logger to a depth of approximately 20 cm before the fire front reached a specific location and removed it after the fire front had passed. By repeating this process multiple times, we collected a sufficient amount of data to demonstrate the hardware's performance for the targeted scientific application.

**Fig. 10:**
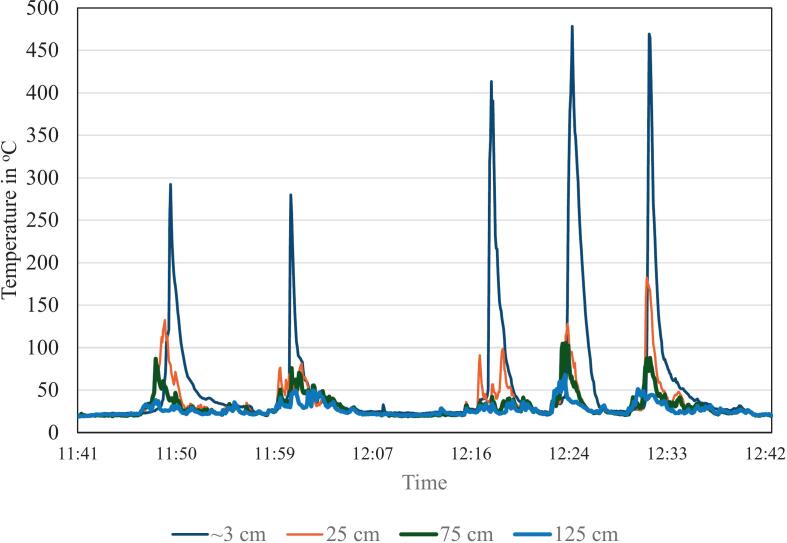
FireLog system readings from a controlled burn (backburn) in an oak woodland in southeastern Nebraska, USA, on March 14, 2024. (T1- ground level (∼3 cm) (in the leaf litter at ground level), T2 – 25 cm, T3 – 75 cm, T4 – 125 cm). Maximum flame heights were approximately <25 cm above the ground at the testing location.

Field testing during a prescribed burn in an oak woodland (Fig. 10) demonstrated that FireLog can withstand temperatures commonly reached during prescribed burns. The maximum temperature was recorded at ground level (∼3 cm), whereas the lowest temperature was noted at a height of 125 cm. The thermocouple at ground level was situated among the leaf litter, and maximum flame heights were <25 cm, which likely accounts for why the highest temperatures were observed at ground level. These temperature-through-time plots can be used to calculate the thermal properties of prescribed burns (Table 2), enabling land managers to tailor prescribed fire ignition methods to achieve the desired fire intensity outcomes.

**Toward a More Integrated Single-Board PCB Design**

The current FireLog prototype utilizes a modular architecture including separate breakout boards for sensing, power management, and communications with wired connections. In the future, we aim to enhance the prototype by utilizing two levels of custom PCB design solutions.

At the first integration level, a custom PCB can be designed to host the existing breakout boards supplied by the OEM (Original Equipment Manufacturer). The Arduino MKR 1010, thermocouple breakout, Arduino MKR MEM board, and real-time clock module can be mounted directly onto female pin headers placed on the custom board. In this configuration, the primary function of the custom PCB is to replace discrete wiring with PCB traces. This approach offers several advantages: it eliminates bulky interconnects and reduces reliance on board-to-board connectors, thereby minimizing size, potential failure points, and assembly complexity. Furthermore, integrated routing of power and signal lines decreases parasitic losses caused by long connectors or separate wiring, which improves overall energy efficiency. An optimized trace layout can also lower resistance, enhance grounding and shielding, and potentially extend battery life.

The level 2 custom PCB is to design a custom PCB including an integrated PCB with the following core ICs: Arduino MKR 1010 [ATSAMD21G18A (ARM Cortex-M0 + MCU) core microcontroller, u-blox NINA-W102 – Wi-Fi/Bluetooth module, battery charging IC (e.g., MCP73831), LDO voltage regulators (3.3 V, 5 V) ], MAX31855 / MAX31856 thermocouple amplifier (SPI) in the thermocouple shield, DS3231 RTC real-time clock with temp compensation in the real time clock breakout, Winbond W25Q16JV (or similar) SPI Flash memory, MicroSD card slot & interface for external storage present in the Arduino MKR MEM shield. Level 2 custom PCB design enables the level 1 custom PCB design advantages with streamlined manufacturing and assembly processes, fewer separate components or subunits can lead to fewer assembly mistakes, easier sealing/potting (for environmental protection), and fewer mechanical failure points. In addition, a cost‐benefit analysis suggests that while initial tooling, design, and prototyping costs may be higher for a single‐board solution, these are amortized over volume. For large-scale deployment (e.g., hundreds or thousands of units), the economies of scale make a single‐board design more cost-effective. Moreover, savings on labor, assembly, enclosure, connectors, and maintenance may offset the increased PCB complexity.

However, there are design challenges, such as thermal management, where a denser board may require a better heat dissipation design. Additionally, noise and interference are concerns, particularly when routing high-speed signals, power circuits, and sensors on the same board, which necessitates careful layout to avoid crosstalk and electromagnetic interference. Flexibility and modularity: although integration gives advantages, modular boards often make it easier to upgrade one subsystem without redesigning the whole device.

## 8 Discussion and future work

We demonstrate a temperature measurement system, FireLog, for use in wildland fires. The FireLog system was developed using open-source hardware and software, incorporating off-the-shelf parts. We also illustrate the deployment protocol and product validation. It is considerably more affordable than other commercial alternatives and easier to assemble, surpassing the capabilities of commercially available data loggers. However, it is important to conduct a dry block temperature sensor calibration to avoid errors due to temperature probe offsets when the dataloggers are put into action in long-term use.

It is also possible to use an even smaller enclosure in the FireLog system than the one mentioned in the bill of materials, with the development of a custom PCB as described in section 8. Size reduction will facilitate deployment in field settings more easily. LiPo batteries with the same energy capacity but a cylindrical shape will further help reduce the system's size.

Although we have not transmitted data through Wi-Fi, as it is not required for the intended experiment, we would like to highlight some relevant past research outcomes that evaluate Wi-Fi, Bluetooth, and LoRa-based communication for the reader's information. High-frequency (5 GHz) Wi-Fi signals attenuate more rapidly than low-frequency (2.4 GHz) Wi-Fi signals, and the attenuation is proportional to the soil moisture content [29,30]. However, 2.4 GHz Wi-Fi successfully transmits data up to 15 cm [29,30]. Compared to Wi-Fi, LoRa communication at 433 MHz frequency can transmit data further down to the soil [31,32]. However, no reliable information was found regarding testing underground Bluetooth communication for signal strength evaluation.

Following the wireless data transmission-related research outcomes, there is significant potential to enhance the current FireLog design. Enabling IoT features will provide a considerable advantage for scientists. However, it is crucial to understand the challenges and opportunities presented by different IoT technologies and protocols, as mentioned by [33]. Our future expectations include testing various IoT technologies in this challenging environment.

## CRediT authorship contribution statement

**Nipuna Chamara:** Writing – review & editing, Writing – original draft, Visualization, Validation, Supervision, Software, Resources, Project administration, Methodology, Investigation, Formal analysis, Data curation, Conceptualization. **Yufeng Ge:** Writing – review & editing, Supervision, Resources, Project administration, Conceptualization. **Sabrina Russo:** Writing – review & editing, Validation, Supervision, Resources, Project administration, Methodology, Investigation, Funding acquisition, Formal analysis, Data curation, Conceptualization.

## Declaration of competing interest

The authors declare that they have no known competing financial interests or personal relationships that could have appeared to influence the work reported in this paper.
